# Vitamin C and Tuberculosis: Examining the Relationship Between Antioxidant Defense and Disease Severity—Preliminary Findings from a Southwestern Romanian Study

**DOI:** 10.3390/jcm13226715

**Published:** 2024-11-08

**Authors:** Ramona Cioboata, Dragos Nicolosu, Andrei-Theodor Balasoiu, Mara Amalia Balteanu, Ovidiu Mircea Zlatian, Andrei Osman, Viorel Biciusca, Eugen-Nicolae Tieranu, Gabriel Florin Razvan Mogos, Alice Elena Ghenea

**Affiliations:** 1 Pneumology Department, University of Medicine and Pharmacy, 200349 Craiova, Romania; ramona_cioboata@yahoo.com (R.C.); biciuscaviorel@gmail.com (V.B.); 2 Pneumology Department, Victor Babes University Hospital, 200515 Craiova, Romania; nicolosud@yahoo.com; 3 Department of Ophthalmology, University of Medicine and Pharmacy of Craiova, 200349 Craiova, Romania; 4 Department of Pulmonology, Faculty of Medicine, Titu Maiorescu University, 031593 Bucharest, Romania; 5 Department of Microbiology, University of Medicine and Pharmacy of Craiova, 200349 Craiova, Romania; ovidiu.zlatian@umfcv.ro (O.M.Z.); gaman_alice@yahoo.com (A.E.G.); 6 Department of Anatomy and Embryology, Faculty of Dentistry, University of Medicine and Pharmacy of Craiova, 200349 Craiova, Romania; andrei.osman@umfcv.ro; 7 Department of Internal Medicine-Cardiology, University of Medicine and Pharmacy Craiova, 200349 Craiova, Romania; eugen.tieranu@umfcv.ro; 8 Department of Surgery, University of Medicine and Pharmacy of Craiova, 200349 Craiova, Romania; gabrielmogos@yahoo.com

**Keywords:** vitamin C deficiency, pulmonary tuberculosis, antioxidant defense

## Abstract

**Background/Objectives:** This study explored the relationship between serum vitamin C levels, antioxidant defense mechanisms, and the severity of pulmonary tuberculosis (TB) among Romanian patients. **Methods:** This study enrolled 53 patients with bacteriologically confirmed pulmonary tuberculosis at Victor Babes University Hospital in Craiova between January 2023 and August 2024. Participants were stratified into two groups based on their serum vitamin C levels: 26 patients with normal levels and 27 patients with low levels. Clinical, demographic, and biological parameters, including inflammatory markers, such as C-reactive protein (CRP) and erythrocyte sedimentation rate (ESR), were assessed at baseline and after 60 days of TB treatment. Serum vitamin C levels were measured using ELISA. The persistence of Mycobacterium tuberculosis (MTB) was evaluated using sputum smear microscopy and culture at baseline and after 2 months of treatment. **Results:** The results showed that patients with low vitamin C levels had significantly higher baseline ESR (92.63 ± 27.69 mm/h) and CRP (43.89 ± 12.00 mg/L) levels compared to those with normal levels (ESR: 65.11 ± 13.27 mm/h, CRP: 31.19 ± 9.76 mg/L). After 60 days, 66.67% of patients with low vitamin C levels remained culture-positive compared to 26.92% in the normal vitamin C group (*p* = 0.004). Multivariate analysis indicated that vitamin C deficiency was significantly associated with a higher TB culture load. **Conclusions**: These findings suggest that vitamin C deficiency may contribute to the persistence of MTB and highlight the potential role of vitamin C supplementation as an adjunct to standard TB treatment, particularly in the context of global efforts to eradicate the disease by 2035.

## 1. Introduction

Tuberculosis (TB) continues to be a major health concern worldwide, with the World Health Organization (WHO) reporting approximately 10 million cases in 2021. Despite active efforts to reduce both the incidence and mortality rates associated with TB, the disease continues to place a substantial burden on healthcare systems globally [1]. Stress responses, including elevated cortisol levels, play a key role in acute infection scenarios [2]. In severe cases, such as those seen in coronavirus disease 2019 (COVID-19) [3], there is growing evidence of hypocortisolemia due to adrenal gland damage, which may manifest as adrenal infarction in up to two-thirds of cases, often requiring prompt glucocorticoid intervention. This highlights the complexity of immune and endocrine interactions in infectious diseases, including tuberculosis.

In the year 2022, a grand total of 7.5 million cases of tuberculosis were detected worldwide. It is estimated that approximately 10.6 million individuals (with a 95% uncertainty interval of 9.9–11.4 million) were infected with TB globally in 2022. Furthermore, TB was accountable for roughly 1.30 million fatalities (with a 95% uncertainty interval of 1.18 to 1.43 million) on a global scale in 2022.

According to data from 2020, Romania has registered the highest incidence of tuberculosis within the European Union/European Economic Area (EU/EEA), accounting for 23.2% of the 33,148 TB cases reported across 29 EU/EEA countries. This significant disparity highlights Romania’s distinct challenge with TB, making it the European country with the highest TB incidence rate at the time [4,5].

The World Health Organization (WHO) has set an ambitious goal to eliminate the tuberculosis epidemic by 2035. This objective includes reducing TB incidence and mortality rates by 90–95%, representing a significant reduction in the global burden of the disease. Achieving this target, however, faces numerous challenges, with one of the most prominent being the lengthy duration of TB treatment. The extended treatment time, which spans six months or more, can lead to poor patient adherence, increased rates of treatment default, and a heightened risk of drug resistance [6,7]. Pulmonary damage caused by TB can lead to reduced lung function and respiratory complications, which not only make the treatment process more complex and prolong the recovery period but also contribute to additional complications that impede successful tuberculosis management [8,9].

The duration of treatment for drug-susceptible TB is six months, and four medications, isoniazid (INH), rifampin (RIF), pyrazinamide (PZA), and ethambutol (EMB), are required to achieve a cure. Patients initially infected with drug-susceptible TB but receiving inadequate treatment may develop multidrug-resistant TB (MDR-TB). MDR-TB treatment, which is resistant to the two most effective first-line TB drugs, isoniazid and rifampin, is prolonged and involves the use of second-line TB drugs with severe side effects [10].

Tuberculosis chemotherapy is challenged by the difficulty of targeting persisters (a subset of Mycobacterium tuberculosis that resists drug and immune responses), which often results in latent infections and the development of drug-resistant strains. However, adjunctive agents that enhance the bactericidal activity of antibiotics can help reduce treatment duration and restore antibiotic efficacy against resistant strains [9].

Chemotherapy could potentially be reduced if medications are available to eliminate TB more quickly. Bactericidal drugs often kill *Escherichia coli* cells by causing the Fenton reaction, which produces highly reactive hydroxyl radicals. Vitamin C, a substance known to cause the Fenton reaction, may have the potential to sterilize both drug-susceptible and drug-resistant *Mycobacterium tuberculosis* cultures [11,12].

Vitamins are well known for their role in boosting the immune system, and several studies have demonstrated their potential to inhibit mycobacterial growth, with vitamins D, C, and E playing a major role in supporting the body’s antioxidant system [13].

Due to a deficiency in the gene encoding gluconolactone oxidase, humans cannot synthesize vitamin C and must obtain it through diet, and available data indicate that a significant proportion of TB patients exhibit a deficiency in vitamin C, also known as ascorbic acid [14].

Vitamin C demonstrated a synergistic effect when combined with the first-line TB drugs isoniazid and rifampin. In two separate experiments involving infected mice, this combination decreased the bacterial load in the lungs more rapidly than isoniazid and rifampin administered separately [15].

High concentrations of vitamin C have been shown to sterilize drug-susceptible, multidrug-resistant (MDR), and extensively drug-resistant (XDR) Mycobacterium tuberculosis, suggesting that vitamin C could help prevent the emergence of drug-resistant TB strains while also influencing susceptibility to TB, disease progression, and plasma vitamin C levels [16].

Several studies have explored the relationship between tuberculosis and vitamin C levels, with Dalton et al. (2014) demonstrating that ascorbic acid has a potent antibacterial effect on Mycobacterium tuberculosis biofilms, suggesting that high concentrations of vitamin C can effectively kill the bacteria responsible for TB [17].

Nikita et al. (2015) support this finding, showing that both drug-sensitive and drug-resistant strains of *Mycobacterium tuberculosis* are inhibited by ascorbic acid in vitro, with higher concentrations leading to no bacterial growth. This suggests a potential role of vitamin C in TB treatment, especially given the challenge of multidrug-resistant MTB strains [18].

Recent studies have highlighted the potential benefits of vitamin C in the management of TB. Specifically, vitamin C has been shown to accelerate the conversion of acid-fast bacilli (AFB) in sputum samples from patients undergoing intensive phase treatment for pulmonary TB [19]. Moreover, its adjunctive use has been associated with improved sputum smear conversion rates when used alongside standard anti-TB therapies (Patel et al., 2024) [20]. These findings suggest that vitamin C may play a beneficial role in enhancing the treatment outcomes for TB patients with TB.

## 2. Materials and Methods

### 2.1. Study Design and Population

This study was conducted prospectively to examine the relationship between disease severity and vitamin C levels in the serum of patients with confirmed tuberculosis.

The present study included 53 patients with bacteriologically confirmed pulmonary tuberculosis who were admitted between January 2023 and August 2024 to the Department of Pneumology at Victor Babes University Hospital in Craiova, Romania.

Participation in the study was voluntary and all patients provided informed consent after being fully informed of the study’s purpose and procedures.

This research was conducted in accordance with the principles outlined in the Helsinki Declaration of 1975 and was approved by the Ethics Review Board of the University Medicine and Pharmacy of Craiova (No.204/05.08.2024) and Victor Babes University Hospital (No. 24616/17.06.2022).

Participants in the study were enrolled following their voluntary informed consent, without any form of political, social, or religious discrimination, and in full accordance with the data protection legislation.

### 2.2. Inclusion and Exclusion Criteria

The stated aim of the inclusion criteria was to recruit patients who were 18 years or older, who provided written informed consent, and who were bacteriologically confirmed to have pulmonary TB. This study only included new cases of pulmonary tuberculosis. TB was defined as having at least one sputum test with a positive acid-fast bacillus test and a positive Xpert MTB RIF test. A new case was that of a patient who had never taken a combination of TB drugs for more than one month.

The exclusion criteria included individuals with significant comorbidities, such as chronic respiratory diseases, including interstitial lung disease, pulmonary fibrosis, bronchiectasis, asthma, chronic obstructive pulmonary disease, and malignancy; those with a past diagnosis of pulmonary tuberculosis; or those who received treatment for latent TB infection, which could significantly impact the research results. We also excluded HIV-positive patients and those lost to follow-up.

Individuals who lacked comprehensive medical records or vital study information, in addition to those receiving treatment that significantly affected their serum vitamin C levels, such as antipsychotic medication or chemotherapy, were excluded [21,22].

### 2.3. Follow-Up

Follow-up evaluations were conducted in accordance with the guidelines of the National Tuberculosis Control Program at two months post-diagnosis to monitor patient progression and assess any alterations in their health status. These evaluations included serial bacteriological and biological examinations, allowing for a comparative analysis across the different time points. It is important to note that vitamin C levels were not measured at the two-month follow-up. This decision was based on literature data indicating that, in the absence of vitamin C supplementation, plasma vitamin C levels do not change significantly in tuberculosis patients during treatment [23,24]. As our patients did not receive vitamin C supplementation, we focused on baseline vitamin C levels as a predictor of treatment outcomes in tuberculosis, which was the primary goal of our study.

During follow-up evaluations, patients were specifically questioned about their smoking behavior to verify any changes. Smoking status remained unchanged for each patient throughout the study, as we closely monitored smoking habits to ensure consistency due to their potential impact on immune function and tuberculosis treatment outcomes.

### 2.4. Data Collection

Upon admission, a comprehensive medical history and physical examination were performed. The results of medical analysis were extracted from the patients’ electronic records.

### 2.5. Laboratory Diagnosis of Tuberculosis

The acid-fast bacilli (AFB) sputum examination was conducted microscopically, and it was analyzed initially and after two months of treatment. Two identification methods were used in this study. Sputum examination was conducted using three specimens of early morning sputum obtained from each patient. The identification of pulmonary tuberculosis (TB) was contingent upon the utilization of the Ziehl–Neelsen staining method, which entailed the detection of AFB through microscopic examination under light/bright-field microscopy and nucleic acid amplification testing. Additionally, a nucleic acid amplification test (GeneXpert, Cepheid, Sunnyvale, CA, USA) using an Xpert MTB RIF kit was used to detect the Mycobacterium tuberculosis complex.

Sputum smear microscopy and culture grading were based on the following criteria: fewer than 30 colonies were classified as positive-1–9 AFB; 30–100 colonies were classified as positive 1+, more than 100 isolated colonies were classified as positive 2+, and uncountable confluent colonies were classified as positive 3+.

### 2.6. Measurement of the Biological Serum Parameters

Venous blood samples (6 mL per patient) were collected to assess the various essential biological parameters. Samples were collected from each patient after an overnight fast and analyzed to determine the complete blood count (leukocytes, hemoglobin, and Platelets), using an Alinity automated blood analyzer (Abbott Diagnostics, Abbott Park, Green Oaks, IL, USA), and to detect hepatic enzymes, urea, serum creatinine, and inflammatory markers such as fibrinogen and C-reactive protein (CRP) using an automated blood chemistry analyzer Architect C8000 (Abbott Diagnostics, Abbott Park, Green Oaks, IL, USA).

### 2.7. Measurement of Serum Levels of Vitamin C

Vitamin C serum levels were analyzed using the Enzyme-Linked ImmunoSorbent Assay (ELISA) method with a vitamin C (Ascorbic Acid) ELISA Kit (Abbexxa, Cambridge, UK). The ELISA was performed according to manufacturer instructions, and for the final reading of the microtiter plates we used a ChroMate 4300 Plate Reader (Awareness Technology, Palm City, FL, USA).

Serum biomarkers were tested before the beginning of treatment and subsequently after a two-month interval.

### 2.8. Statistical Analysis

The sample size calculation showed that to detect a 40% difference in the TB conversion rate between patients with normal (supposed to be 30%) and low (supposed to be 70%) vitamin C levels, with a significance level of 0.05 and a power of 80%, the required sample size was 21 patients in each group.

Data were analyzed after being imported into STATA 17.0 SE statistical software (StataCorp Ltd., College Station, TX, USA). Descriptive statistics were used to summarize demographic and clinical characteristics of the study participants. Continuous variables were expressed as mean ± standard deviation (SD). Categorical variables were presented as frequencies and percentages. Comparisons between groups (e.g., patients with normal and low vitamin C levels) were performed using an independent t-test for continuous variables. For categorical variables, Fisher’s exact test was used. The persistence of culture positivity after 60 days of treatment was analyzed using risk ratios (RRs) with 95% confidence intervals (CIs) and a Chi2 statistical test. Multivariate logistic regression was employed to adjust for potential confounding factors such as age, smoking status, and BMI. In addition to traditional statistical analyses, Structural Equation Modeling (SEM) was employed to explore the relationships between vitamin C levels and the persistence of tuberculosis (TB) infection while accounting for multiple covariates.

A longitudinal analysis was conducted to evaluate changes in clinical parameters (e.g., ESR and CRP) over time. Repeated measures ANOVA and random-effects modeling were used to assess the interaction between time and vitamin C levels. A *p*-value of <0.05 was considered statistically significant for all analyses. All statistical tests were two-tailed.

## 3. Results

Initially, 61 individuals diagnosed with pulmonary tuberculosis were screened for eligibility. Of these, 53 met the predetermined inclusion criteria and were subsequently enrolled in the study.

### 3.1. Vitamin C Levels in the Studied Patients

A total of 53 participants (14 females and 39 males) were included in the study. Vitamin C levels varied significantly among the participants, with levels ranging between 0.40 and 2.32 µg/mL and with an average of 0.68 µg/mL. The standard deviation was 0.42 µg/mL, showing some variability in vitamin C levels within the sample. We divided the patients in one test group with low vitamin C serum levels and one control group with normal vitamin C serum levels, according to the manufacturer instructions, which state that for this ELISA kit the normal range of vitamin C serum levels is 0.49–40 µg/mL. We chose the threshold of 0.92 µg/mL, which is recommended by “The Dietary Guidelines for Americans”, nutrition guidance for people across lifespans published every 5 years by the USA Office of Disease Prevention and Health Promotion [25].

Specifically, 26 individuals (49.06%) had normal vitamin C levels, while 27 (50.94%) had low vitamin C levels.

### 3.2. Biological Parameter Distribution According to Serum Vitamin C Levels

The study revealed a notable predominance of males, comprising 73.58% of the total population. The mean age of individuals with low vitamin C (43.15 years) was higher than that of those with normal levels (39.04 years). The age group distribution indicated a significant representation of individuals aged 18–40 years. Both demographics maintained a normal body mass index (BMI), although those with normal vitamin C levels exhibited slightly higher mean BMIs. Notably, 74.07% of the individuals with low vitamin C levels were smokers, suggesting that smoking may contribute to vitamin C deficiency (Table 1).

The initial parameters were compared based on vitamin C levels, and significant differences were observed for several markers. Subjects with lower vitamin C levels exhibited higher initial ESR (92.63 ± 27.69 mm/h) and CRP (43.89 ± 12.00 mg/L) levels compared to those with normal vitamin C levels (ESR: 65.11 ± 13.27 mm/h, CRP: 31.19 ± 9.76 mg/L), indicating a potential correlation between vitamin C deficiency and inflammatory response (Table 2).

In females (27.88 ± 5.62 mg/dL) compared to males (32.17 ± 9.78 mgIn the normal vitamin C group, significant gender differences were observed in hemoglobin, leukocytes (WBCs), and urea levels. Hemoglobin levels were significantly lower in females (11.80 ± 1.30 g/dL) compared to males (13.03 ± 1.46 g/dL) (*p* = 0.001). Leukocyte counts were also significantly lower in females (7779 ± 1288/mL) than in males (6654 ± 1501 × 10^3^/mL) (*p* = 0.003). Additionally, urea levels were lower /dL) (*p* = 0.043).

In the low vitamin C group, there were no statistically significant differences in hemoglobin, leukocytes (WBCs), or urea levels between males and females.

### 3.3. Persistence Rates of MTB

In the group with normal vitamin C levels, the data demonstrated a statistically significant improvement in microscopic acid-fast bacilli (AFB) load after 60 days of treatment. The overall reduction in total microscopy-positive cells from 92.31% to 38.46% indicates that the treatment substantially decreased bacterial presence.

However, the persistence of the 1+ category (34.61%) suggests that while some patients exhibit improvement, a subset still harbors detectable AFB. Consistent with the microscopic results, the culture results demonstrated a statistically significant improvement, with the proportion of culture-negative patients increasing from 0% at baseline to 73.08% post treatment. Of particular note are the negative results for MTB both by culture and microscopy in patients with cultures of 2+ and 3+ (Table 3).

Table 4 presents the microscopic acid-fast bacilli (AFB) load and culture results before and after 60 days of treatment in 27 patients diagnosed with pulmonary tuberculosis (TB) and low serum vitamin C. At baseline, all 27 patients exhibited positive microscopic results, indicating significant AFB loads, predominantly in the 2+ and 3+ categories. However, the microscopic persistence of AFB in 51.85% of the patients, particularly in the lower ranges of AFB (1–9 and 1+), suggests that a substantial proportion of individuals have not yet fully eliminated MTB.

The initial culture results indicated that all the patients were culture-positive, which is a standard indicator of viable MTB. After 60 days, there was a reduction in the total culture positivity to 66.67%.

Overall, 66.67% of patients with low vitamin C levels remained culture-positive after 60 days of treatment, compared to 26.92% in the normal vitamin C group (risk ratio = 2.48, Chi2 *p* = 0.004).

At baseline, none of the patients were microscopy-negative for acid-fast bacilli (AFB) (Table 5). Among male patients, those with low vitamin C levels exhibited higher microscopic AFB loads compared to those with normal vitamin C levels. Specifically, in males, in the low vitamin C group 42.85% of patients were in the 3+ microscopy category, compared with 11.11% in the normal vitamin C group. Among female patients, a similar trend was observed, as 83.34% of those with low vitamin C levels were in categories 2+ and 3+, compared with 37.50% with normal vitamin C levels.

Following 60 days of standard TB treatment, an increased proportion of patients became microscopy-negative, particularly in the normal vitamin C group. Among males, 42.85% of patients with low vitamin C levels were microscopy-negative, compared to 55.55% in the normal vitamin C group (risk ratio = 1.03, Chi2 *p* = 0.921). In females, 66.67% with low vitamin C levels became microscopy-negative versus 75.00% in the normal vitamin C group (risk ratio = 1.33, Chi2 *p* = 0.733). These findings suggest that low vitamin C levels are associated with a higher risk of microscopic AFB persistence among female patients compared to male patients, as indicated by a greater risk ratio in females (1.33) than in males (1.03).

At baseline, all patients were culture-positive for AFB. In male patients, higher culture AFB loads were observed in those with low vitamin C levels. In the low vitamin C group, 33.33% were in the 3+ culture category, and only 5.55% were in the normal vitamin C group. Among females, the percentages were 16.67% for the low vitamin C group and 12.50% for the normal vitamin C group.

After 60 days of treatment, a significant difference was observed between the low and normal vitamin C groups in terms of culture conversion. Among males, 71.42% of those with low vitamin C levels remained culture-positive at the 1+ level, compared to 27.78% of those in the normal vitamin C group (risk ratio = 2.57, Chi2 *p* = 0.007). In females, 50.00% of patients with low vitamin C levels remained culture-positive at the 1+ level versus 25.00% of those in the normal vitamin C group (risk ratio = 2.00, Chi2 *p* = 0.334).

**Figure 1:**
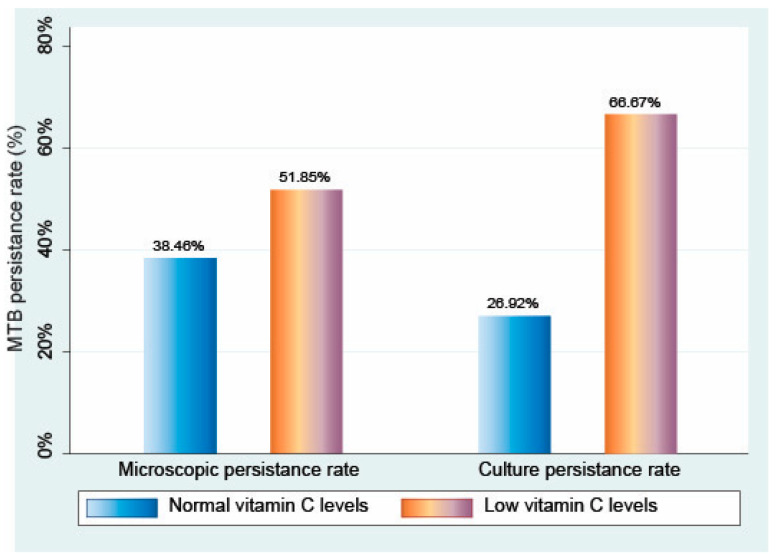
Comparison of persistence rate of tuberculosis in patients with normal levels of vitamin C compared with patients with low levels of vitamin C.

Figure 1 summarizes the persistence rates of *Mycobacterium tuberculosis* in both the groups. In the group with normal vitamin C levels, persistence was 38.46% in microscopy and 26.92% in culture, compared with 51.85% in microscopy and 66.67% in culture in the group with low vitamin C levels.

**Figure 2:**
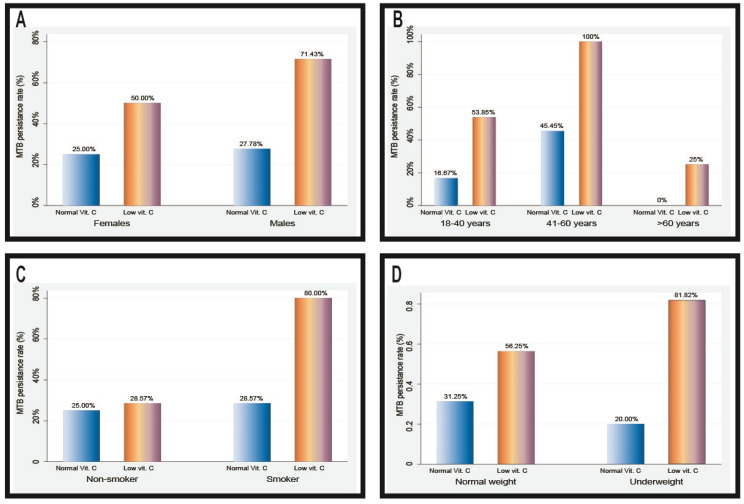
MTB persistence rate (%) stratified by patient gender (**A**), age group (**B**), smoking status (**C**), and weight (**D**).

Among individuals with low vitamin C levels, the persistence rate of *Mycobacterium tuberculosis* (MTB) was higher in males (71.43%) than in females (50.00%). The highest persistence rate was noted in the 41–60-year age group, with smokers in the low vitamin C group demonstrating a significantly higher rate (80.00%) than non-smokers (28.57%). Moreover, underweight patients with low vitamin C levels exhibited a higher persistence rate (81.82%) than those with normal weight (56.25%) (Figure 2).

### 3.4. Evolution of Biological Parameters After TB Treatment

**Figure 3:**
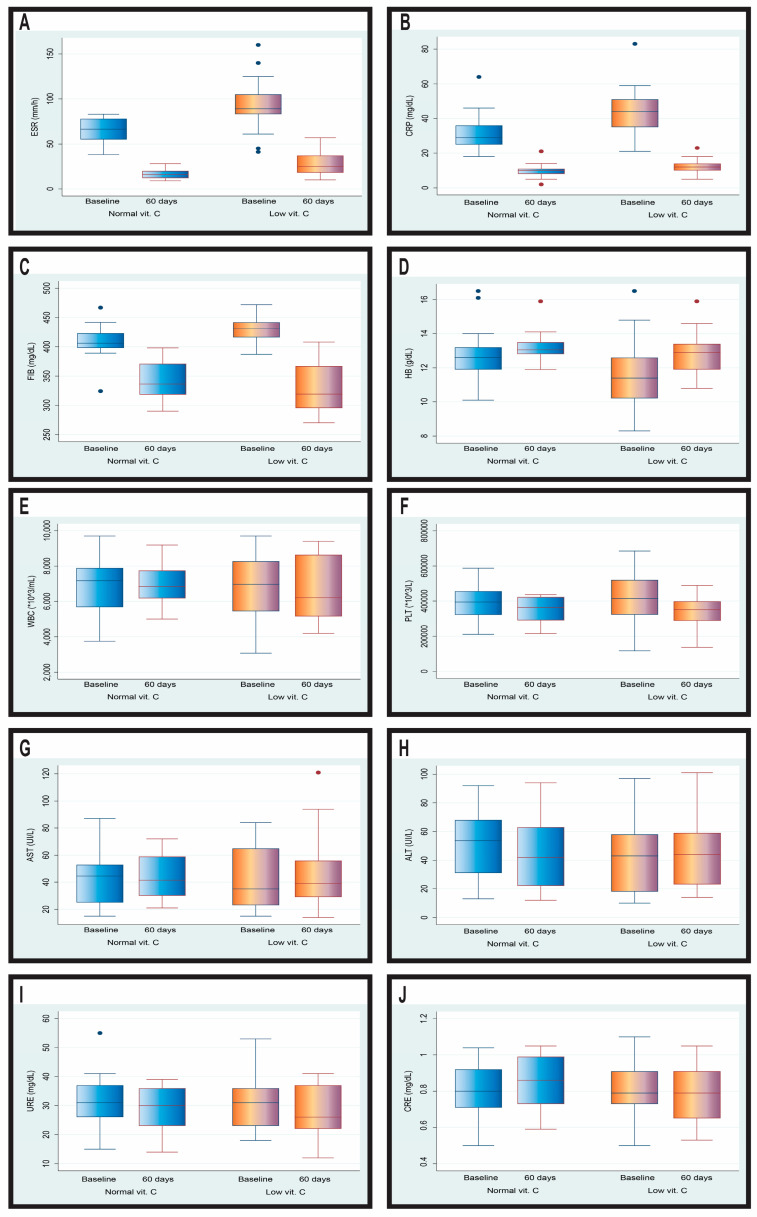
Temporal changes in the biological parameters of MTB patients after 60 days of treatment stratified by vitamin C levels. (**A**): ESR (erythrocyte sedimentation rate); (**B**): CRP (C reactive protein); (**C**): FIB (fibrinogen); (**D**): HB (hemoglobin); (**E**): WBC (white blood cells); (**F**): PLT (platelets); (**G**): AST (aspartate aminotransferase); (**H**): ALT (alanine aminotransferase); (**I**): URE (urea); (**J**): CRE (creatinine).

Table 6 and Figure 3 summarize the alterations in various parameters based on vitamin C levels. It is evident that patients with low vitamin C levels demonstrated more substantial changes across several health indicators than those with normal vitamin C levels.

With respect to temporal changes, statistically significant differences were observed in several parameters between groups. Specifically, patients with low vitamin C levels exhibited a greater reduction in ESR (−58.56 ± 19.07 mm/h) compared to those with normal vitamin C levels (−48.15 ± 13.94 mm/h). Additionally, the reduction in CRP was more substantial in the low vitamin C group (−29.44 ± 12.34 mg/L) than in the normal vitamin C group (−21.54 ± 9.21 mg/L). A more pronounced reduction in fibrinogen level was observed in the low vitamin C group (−99.7 ± 48.05) than in the normal group (−65.73 ± 49.12), further supporting the trend of reduced inflammation.

An increase in hemoglobin levels was observed, with the low vitamin C group demonstrating a higher increase, while the white blood cell count presented a slight increase in the low vitamin C group (44.78 ± 1754.86), in contrast to a minor decrease in the normal vitamin C group (−68.27 ± 1366.38). However, owing to the high standard deviations, these changes were not statistically significant; both groups exhibited a decrease, but it was more substantial in the low vitamin C group (−78,092.59 ± 119,134.5) than in the normal group (−46,038.46 ± 112,753.0).

Both groups demonstrated minor changes in ALT and AST levels, indicating a stable overall liver function. Urea levels decreased in both groups, suggesting stable kidney function (Table 6).

### 3.5. Multivariate Analysis

Longitudinal random-effects regression analysis was used to investigate the factors influencing the persistence of culture-positive results after 60 days of anti-TB treatment. Table 7 summarizes the results of the multivariate analysis. Each variable’s coefficient indicates its association with the outcome, accompanied by standard errors, *z*-values, *p*-values, and 95% confidence intervals.

A coefficient of 3.83 (*p* = 0.034) indicated that low levels of vitamin C were significantly associated with a higher TB culture load over time. The positive coefficient suggests that lower vitamin C levels correlate with an increase in the TB culture load.

The coefficient of 0.39 is not statistically significant (*p* = 0.848), indicating no evidence that gender affects the TB culture load over time. While the coefficient of 2.88 is positive, suggesting that smokers may have higher TB culture loads, it is not statistically significant (*p* = 0.134); therefore, the effect is uncertain. The coefficient of 0.71 is also not significant (*p* = 0.698), indicating no evidence that being underweight influences the TB culture load (Table 7).

### 3.6. Generalized Structural Equation Modeling

**Figure 4:**
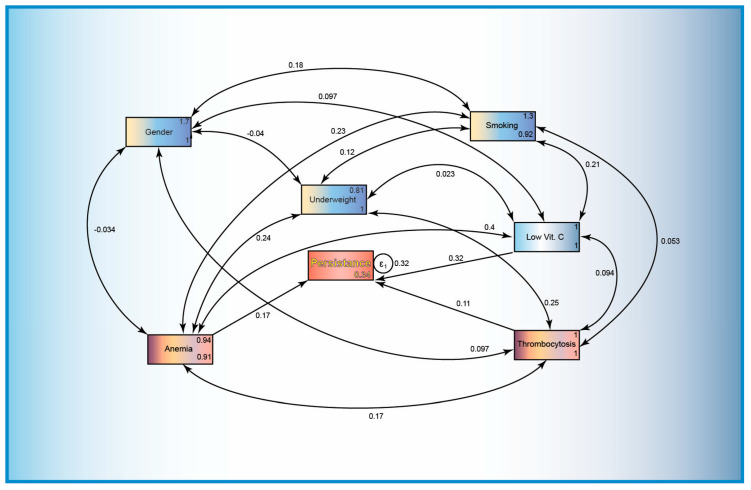
Structural equation model of the persistence of MTB infection using observed variables such as demographic factors (gender, smoking status, and underweight status) and biological parameters (anemia, thrombocytosis, and low serum levels of vitamin C). The numbers on the arrows represent the relative influence of one variable on another or covariance. In the variable squares, the upper right number is the variance of the variable, while the lower right number is the variance of the residuals (the amount of variable variance not explained by the model) where ε1 is the error term in the model.

The structural equation model we developed (Figure 4) assessed the persistence of MTB infection as the dependent variable, with several independent variables including vitamin C levels, anemia, thrombocytosis, patient gender, smoking status, and underweight status. The model demonstrated that lower vitamin C levels were significantly associated with an increased likelihood of MTB persistence at 60 days (coefficient 0.3188; *p* = 0.013). Specifically, for each unit decrease in vitamin C levels, the probability of TB persistence increased by approximately 32%.

Covariates had varying effects on persistence. Anemia (coefficient: 0.1726, *p* = 0.197) indicated a potential association with TB persistence, while thrombocytosis (coefficient: 0.1117, *p* = 0.368) did not emerge as a strong predictor of MTB persistence in this model.

The means of exogenous variables, such as low vitamin C levels and patients’ gender, smoking status, underweight status, thrombocytosis, and anemia, provide the average levels or prevalence of these factors in the study population. In terms of notable significance, a mean gender of 1.67 indicates that the gender distribution has a significant impact on the persistence of MTB, with males possibly being more affected; smoking status with a mean of 1.34 and a significant *p*-value (0.000) appears to have a strong influence on MTB persistence; underweight status with a mean of 0.81 and a significant *p*-value (*p* = 0.000) suggests that being underweight is a significant factor in the context of MTB persistence.

Covariances between low vitamin C levels and anemia (covariance coefficient: 0.3980, *p* = 0.001) were significant, suggesting that these two conditions tend to occur together and may jointly influence MTB persistence. There was also a significant positive covariance (0.2548, *p* = 0.047) between underweight status and thrombocytosis, indicating that these two conditions also tended to co-occur, which might affect their impact on TB persistence. Other covariances, such as those between smoking and anemia or between gender and other variables, were not statistically significant, suggesting weaker relationships.

For endogenous (dependent) persistence, a single number represents the variance in the residuals (error term), indicating how much of the variance in persistence is not explained by the predictors in the model. In other words, only 34% of the persistence variable is unexplained and 66% is explained by the statistical model.

## 4. Discussion

To the best of our knowledge, this research is one of the earliest investigations into vitamin C levels and their relationship with pulmonary tuberculosis severity in the Romanian population, providing novel insights into a potential complementary treatment approach.

Vitamin C has been investigated for its potential role in the treatment of tuberculosis (TB). Research suggests that vitamin C supplementation may enhance the efficacy of anti-tuberculosis drugs (ATD), improve chest X-ray images in pulmonary TB patients during the intensive phase of treatment (Jefri et al., 2020) [19], and increase sputum conversion rates, indicating an improvement in the healing process of TB patients [20].

Our results indicate that vitamin C levels varied significantly among the participants, with levels ranging between 0.4 and 2.32 µg/mL and an average of 0.68 µg/mL. The standard deviation was 0.42 µg/mL, showing some variability in vitamin C levels within the sample.

Importantly, the study revealed that 74.07% of individuals with low vitamin C levels were smokers, highlighting a clear association between smoking status and vitamin C deficiency. This correlation reinforces the findings of previous studies, which suggest that smoking not only increases oxidative stress in the body by generating free radicals, weakening antioxidant defenses, and causing DNA, protein, and lipid damage [26,27], but may also impair the absorption and utilization of essential nutrients, such as vitamin C. As such, the data further bolster the argument that interventions aimed at increasing vitamin C intake and addressing smoking behaviors may be particularly crucial for older male populations at risk of undernutrition. A 2017 study conducted on 51 non-smokers and 20 smokers indicated that smokers in this cohort were predominantly male. Furthermore, smokers were significantly older than non-smokers (*p* < 0.0003), implying patterns of smoking behavior that may be age-dependent [28].

The data showed that individuals with low vitamin C levels had a mean age of 43.15 years, higher than those with normal levels (39.04 years), supporting the assertion from the literature that older individuals frequently exhibit associations with undernutrition and hypovitaminosis C. This correlation suggests that older age may be a key factor influencing dietary habits, absorption, and overall nutritional status, including vitamin C levels. The significant representation of individuals aged 18–40 years indicates a potential divergence in lifestyle choices and nutritional uptake among younger adults who may not yet experience the same impacts of aging-related nutritional deficiencies [28].

Additionally, while both groups maintained a normal body mass index (BMI), the slightly higher mean BMI observed in the normal vitamin C group suggests that proper nutrition, including adequate vitamin C levels, supports better overall body composition. In contrast, findings from the literature that smokers typically demonstrate lower BMI levels reinforce the notion that smoking may interfere with nutritional status, potentially leading to weight loss or insufficient caloric intake and affecting vitamin C levels [28]. In 2017, Pearson et al. observed a higher prevalence of hypovitaminosis C and deficiency among individuals with lower socioeconomic status and current smokers [29,30].

Baseline biological parameters were compared based on vitamin C levels, and notable differences were observed in several markers. Patients with lower vitamin C levels had higher initial ESR (92.63 ± 27.69 mm/h) and CRP (43.89 ± 12.00 mg/L) levels than those with normal vitamin C levels (ESR: 65.11 ± 13.27 mm/h, CRP: 31.19 ± 9.76 mg/L), suggesting a possible association between vitamin C deficiency and inflammatory response. The higher CRP levels in the low vitamin C group can be explained by increased oxidative stress but also by the higher number of smokers in this group, because smoking can be associated with higher CRP levels [28] as well as with higher ESR levels [31]. No significant differences were observed in the WBC count, PLT count, and serum levels of alanine aminotransferase, aspartate aminotransferase, urea, or creatinine.

In the low vitamin C group, typical differences in hemoglobin, leukocytes, and urea levels between men and women seem to diminish, likely because vitamin C deficiency impacts these physiological processes in both sexes similarly. Vitamin C is essential for efficient iron absorption and red blood cell production, so when levels are low, both men and women can experience reductions in hemoglobin due to impaired iron utilization. This deficiency potentially narrows the usual gender gap in hemoglobin levels, as both sexes face limitations in red blood cell production without sufficient vitamin C.

Leukocyte counts, too, are influenced by vitamin C due to its role in immune function. Vitamin C supports white blood cell production and shields these cells from oxidative stress. With insufficient vitamin C, immune function may weaken uniformly across both sexes, reducing the typical difference in leukocyte levels seen in normal conditions. The lack of sufficient antioxidant support could lead to oxidative stress in immune cells for both men and women, leveling out their white blood cell counts.

Finally, urea levels, influenced by muscle mass and protein metabolism, may also converge under low vitamin C conditions. When vitamin C is deficient, muscle breakdown can increase, raising urea levels for both men and women. Additionally, vitamin C supports kidney health, so a deficiency could impair kidney function in both sexes, reducing the usual differences in urea processing. Together, these effects suggest that vitamin C deficiency creates physiological stresses that override the typical gender differences observed when vitamin C levels are normal.

Significant differences were observed in several parameters between the groups over time. For instance, patients with low vitamin C levels showed a greater reduction in inflammatory markers compared to those with normal vitamin C levels, such as ESR (−65.63 ± 19.44 mm/h vs. −48.15 ± 13.94 mm/h), CRP (−32.0 ± 12.23 mg/L vs. −21.54 ± 9.21 mg/L), and FIB (−99.7 ± 48.05 vs. −65.73 ± 49.12). Patients with low vitamin C levels likely have higher baseline ESR values, indicating greater initial inflammation. Vitamin C has anti-inflammatory properties, and its deficiency could exacerbate inflammatory processes, leading to an elevated ESR. Thus, a larger decrease in ESR after treatment may reflect a greater initial inflammatory burden being reduced but not fully resolved. The treatment may have been less effective in completely normalizing the ESR in vitamin C-deficient patients, as adequate vitamin C is necessary for optimal healing and recovery. Despite the larger decrease in ESR without sufficient vitamin C, these patients may still be dealing with residual inflammation, reflected in their higher final ESR values.

Importantly, low levels of vitamin C were also observed in some TB patients and, in a sizable population-based cohort study, adequate dietary intake of vitamin C has been suggested to protect against developing active TB in current smokers [32].

Vitamin C has been shown to enhance the elimination of both drug-sensitive and treatment-resistant *Mycobacterium tuberculosis* in vitro. Research conducted by Vilchèze et al. showed that vitamin C initiates the Fenton reaction, which facilitates the transformation of ferric ions into ferrous ions in oxygenated environments [33]. This process results in the generation of reactive oxygen species (ROS), including superoxide, hydrogen peroxide, and hydroxyl radicals [33,34]. The formation of these radical species induces substantial damage to MTB DNA and lipids, effectively inhibiting growth and reproduction. These findings suggest that reduced vitamin C levels may be associated with the persistence of MTB and indicate the potential of vitamin C as an adjuvant therapy for tuberculosis treatment, particularly in addressing challenges related to drug resistance.

Our findings indicated that among individuals with low vitamin C levels, the persistence rate of *Mycobacterium tuberculosis* (MTB) is higher in males (71.43%) than in females (50.00%), which aligns with the existing literature that shows gender differences in TB susceptibility and treatment outcomes. Studies have consistently reported that male patients are more likely to experience poorer health outcomes and disease persistence, potentially driven by biological, environmental, and behavioral factors [35].

At baseline, patients with low vitamin C levels exhibited higher microscopic and culture AFB loads compared to those with normal vitamin C levels. Specifically, a greater proportion of patients in the high-AFB-load categories (2+ and 3+) were found in the low vitamin C group. This suggests that vitamin C deficiency may be linked to a higher bacterial burden at the onset of the disease.

Vitamin C is known to play a crucial role in the immune system, particularly in enhancing the function of phagocytes and the oxidative killing of pathogens. A deficiency in vitamin C could impair these immune functions, leading to an inadequate initial response to *Mycobacterium tuberculosis* infection and allowing the bacteria to proliferate more extensively.

The fact that 66.67% of patients with low vitamin C levels remained culture-positive compared to only 26.92% in the normal vitamin C group is clinically significant. Culture positivity after two months of treatment is associated with a higher risk of treatment failure and relapse [36]. This finding underscores the potential impact of vitamin C levels on treatment outcomes.

Notably, the higher persistence rate of MTB in smokers (80.00%) compared to non-smokers (28.57%) among individuals with low vitamin C status is supported by the findings of Khameneh et al. (2016), who demonstrated that smoking could contribute to oxidative stress and impair immune responses, thereby increasing the probability of TB persistence [33,34].

Furthermore, the data indicated that underweight patients with low vitamin C levels exhibited an 81.82% persistence rate, which is substantially higher than the 56.25% observed in those with normal weight. This observation is consistent with other studies that have reported that malnutrition and vitamin C deficiency in TB patients are associated with an elevated risk of developing the disease and experiencing severe outcomes [35,37,38].

The results of the longitudinal random-effects regression analysis assessing the factors influencing the persistence of culture-positive results in patients undergoing anti-TB treatment revealed several notable associations. The analysis indicated that low levels of vitamin C were significantly associated with an increased TB culture load over time, with a coefficient of 3.83 (*p* = 0.034). This highlights the critical association between vitamin C levels and persistence, indicating that lower vitamin C levels could be a potential risk factor for prolonged MTB persistence. This finding is consistent with other studies that have drawn similar conclusions regarding the role of vitamin C in modulating the immune response to tuberculosis. For instance, Khameneh et al. (2016) demonstrated that vitamin C could enhance bactericidal activity against *Mycobacterium tuberculosis*, suggesting that deficiencies in this vitamin may lead to poorer treatment outcomes and the persistence of infection [33,34].

The positive coefficient for smoking status (2.88, *p* = 0.134) suggests that smokers may exhibit higher TB culture loads. Although this finding was not statistically significant, it aligns with studies suggesting that smokers exhibit higher TB culture loads due to weakened immune responses, increased bacterial burden, and systemic inflammation, leading to negative treatment outcomes and higher susceptibility to TB infection.

The statistically insignificant coefficient for underweight status (0.71, *p* = 0.698) indicates that nutritional condition does not appear to have a substantial impact on the TB culture load. Similarly, the coefficient for gender (0.39, *p* = 0.848) suggests that gender does not significantly affect TB culture load. A study by Chi C. Leung et al. (2015) investigated treatment outcomes and found that while gender differences exist in susceptibility and health-seeking behaviors, these do not consistently translate into differences in culture positivity or treatment responses [39,40].

The findings of the structural equation model indicated a significant association between lower vitamin C levels and increased persistence of *Mycobacterium tuberculosis* (MTB) infection, with a coefficient of 0.3188 (*p* = 0.013). This result elucidates the critical role of vitamin C as an essential micronutrient for immune function, emphasizing its potential influence on tuberculosis infection control and management. The observed increase of approximately 32% in the probability of TB persistence with each unit decrease in vitamin C levels corroborates previous studies that have demonstrated the antioxidant properties of vitamins and their role in enhancing immune responses against various pathogens, including MTB.

Research conducted by Susanto et al. (2018) revealed that after eight weeks, the treatment group achieved a 100% conversion rate, while the control group reached 83.9% (*p* = 0.02). This notable difference suggests that vitamin C supplementation significantly enhances the recovery process for tuberculosis patients throughout the treatment period [24].

Further, in the structural equation model, anemia and thrombocytosis showed potential associations, although these were not statistically significant. The covariances indicate important relationships between vitamin C and anemia as well as between underweight status and thrombocytosis, which might further influence the outcomes.

### Limitations

This study has several limitations that must be acknowledged. First, the sample size was relatively small, with only 53 patients included. This limits the generalizability of the findings and may reduce the statistical power of the study, particularly when analyzing subgroups such as smokers, patients of different ages, or those with comorbidities. Large-scale studies are required to validate these preliminary findings.

Second, while vitamin C levels were measured and analyzed in relation to tuberculosis treatment outcomes, other potential confounding factors, such as dietary intake, nutritional status beyond vitamin C, or other micronutrients, were not thoroughly assessed. This limits our ability to fully understand the sole role of vitamin C in TB persistence.

Additionally, the study was conducted in a single medical center in southwestern Romania, which may have introduced geographical and demographic biases. Factors such as access to local healthcare, socioeconomic conditions, and environmental influences on vitamin C levels and tuberculosis progression were not considered.

Lastly, the study design is observational, which limits the ability to infer causal relationships between vitamin C levels and tuberculosis outcomes. Randomized controlled trials with vitamin C supplementation as an intervention would be necessary to establish causality and determine the optimal vitamin C dosing in TB management.

## 5. Conclusions

This study provides evidence that low levels of vitamin C are significantly associated with increased persistence of tuberculosis. Vitamin C deficiency was correlated with higher inflammation markers, such as ESR and CRP, and poorer treatment outcomes, emphasizing the importance of adequate vitamin C intake in improving TB treatment efficacy. These findings suggest that vitamin C supplementation could enhance the bactericidal effects of anti-TB drugs, potentially reducing treatment duration and preventing the emergence of drug-resistant strains.

This study highlights the importance of addressing nutritional deficiencies, particularly among at-risk populations, such as smokers, older individuals, and those with poor nutritional status, to improve TB treatment outcomes. Future research should focus on larger cohort studies and clinical trials to further substantiate the role of vitamin C as an adjunct therapy in TB treatment and to explore optimal supplementation strategies for different patient populations.

## Figures and Tables

**Table 1:** Demographic parameters of the patients stratified according to serum levels of vitamin C. BMI = body mass index.

Parameter	Normal Vitamin C (*n* = 26)	Low Vitamin C (*n* = 27)	Total (*n* = 53)
Gender ^NS^			
Males	18 (69.23%)	21 (77.78%)	39 (73.58%)
Females	8 (30.77%)	6 (22.22%)	14 (26.42%)
Age (years) ^NS^	39.04 ± 14.76	43.15 ± 14.05	41.13 ± 14.41
Age group ^NS^			
18–40 years	12 (46.15%)	13 (48.15%)	25 (47.17%)
41–60 years	11 (42.31%)	10 (37.04%)	21 (39.62%)
>60 years	3 (11.54%)	4 (14.81%)	7 (13.21%)
BMI ^NS^	19.75 ± 2.29	19.55 ± 2.10	19.64 ± 2.18
Smoking ^NS^			
Smokers	14 (53.85%)	20 (74.07%)	34 (64.15%)
Non-smokers	12 (46.15%)	7 (25.93%)	19 (35.85%)

^NS^: non-significant *p* value (>0.05).

**Table 2:** Baseline biological parameters stratified according to serum levels of vitamin C. ESR, Erythrocyte Sedimentation Ratio. CRP = C-reactive protein. FIB = fibrinogen. HB = hemoglobin. WBC = white blood cell. PLT = Platelet. GOT, Glutamate Oxaloacetate Transaminase. GPT = Glutamate Pyruvate Transaminase. URE = urea. CRE = creatinine.

Parameter	Normal Vitamin C Females	Normal Vitamin C Males	Normal Vitamin C Total	Low Vitamin C Females	Low Vitamin C Males	Low Vitamin C Total
ESR (mm/h) *	61.00 ± 15.60	66.94 ± 12.13	65.12 ± 13.27	90.17 ± 20.13	93.33 ± 29.89	92.63 ± 27.69
CRP (mg/dL) *	28.38 ± 7.44	32.44 ± 10.58	31.19 ± 9.76	46.83 ± 18.39	43.05 ± 9.96	43.89 ± 12.00
Fibrinogen (mg/dL) *	414.50 ± 23.59	406.44 ± 24.79	408.92 ± 24.25	428.00 ± 9.03	430.52 ± 20.49	429.96 ± 18.43
Hemoglobin (g/dL) *	11.80 ± 1.30	13.03 ± 1.46	12.65 ± 1.50 ^¥^	11.43 ± 2.16	11.53 ± 1.81	11.51 ± 1.85
WBC (×10^3^/mL) ^NS^	7779 ± 1288	6654 ± 1501	7000 ± 1509 ^¥^	7286 ± 1617	6589 ± 1747	6744 ± 1714
Platelet (×10^3^/mL) ^NS^	397,875 ± 122,648	397,444 ± 95,323	397,576 ± 101,935	400,000 ± 83,500	421,761 ± 163,704	416,925 ± 148,460
GOT (UI/L) ^NS^	40.25 ± 17.29	47.67 ± 21.93	45.38 ± 20.57	41.50 ± 24.10	43.57 ± 23.75	43.11 ± 23.37
GPT (IU/L) ^NS^	45.38 ± 26.68	53.39 ± 21.32	50.92 ± 22.86	37.33 ± 22.94	44.33 ± 28.19	42.78 ± 26.86
Urea (mg/dL) ^NS^	27.88 ± 5.62	32.17 ± 9.78	30.85 ± 8.83 ^¥^	27.83 ± 12.59	31.52 ± 8.18	30.70 ± 9.19
CRE (mg/dL) ^NS^	0.78 ± 0.16	0.82 ± 0.16	0.81 ± 0.15	0.79 ± 0.15	0.82 ± 0.14	0.81 ± 0.14

*: significant difference (*p* ≤ 0.05) between groups of patients with normal and low vitamin C levels. ^¥^: significant difference (*p* < 0.05) between groups of patients with normal and low vitamin C levels. ^NS^: non-significant difference (*p* > 0.05) between groups of patients with normal and low vitamin C levels.

**Table 3:** Microscopic AFB load and culture results in the studied patients at baseline and after 60 days of treatment. AFB = acid-fast bacilli in patients with normal serum vitamin C.

Parameter	Baseline (*n* = 26)	60 Days of Treatment (*n* = 26)
Microscopic AFB load		
Negative	2 (7.69%)	16 (61.54%)
1–9 AFB	2 (7.69%)	1 (3.85%)
1+	9 (34.61%)	9 (34.61%)
2+	11 (42.31%)	0 (0%)
3+	2 (7.69%)	0 (0%)
Microscopy+	24 (92.31%)	10 (38.46%)
Culture		
Negative	0 (0%)	19 (73.08%)
1+	12 (46.15%)	7 (26.92%)
2+	12 (46.15%)	0 (0%)
3+	2 (7.69%)	0 (0%)
Culture+	26 (100%)	7 (26.92%)

**Table 4:** Microscopic AFB load and culture results in studied patients at baseline and after 60 days of treatment. AFB = acid-fast bacilli in patients with low serum vitamin C.

Parameter	Baseline (*n* = 27)	60 Days of Treatment (*n* = 27)
Microscopic AFB load		
Negative	0 (0.00%)	13 (48.15%)
1–9 AFB	1 (3.70%)	3 (5.66%)
1+	3 (5.66%)	10 (37.04%)
2+	13 (48.14%)	1 (3.70%)
3+	10 (37.04%)	0 (0%)
Microscopy+	27 (100%)	14 (51.85%)
Culture		
Negative	0 (0.00%)	9 (33.33%)
1+	5 (18.52%)	18 (66.67%)
2+	14 (51.85%)	0 (0.00%)
3+	8 (29.63%)	0 (0.00%)
Culture+	27 (100%)	18 (66.67%)

**Table 5:** Microscopic AFB load and culture results in the studied patients, stratified by gender. AFB = acid-fast bacilli in patients with low serum vitamin C.

3	Males						Females					
	Baseline			60 Days of Treatment			Baseline			60 Days of Treatment		
	Low Vitamin C (*n* = 21, 100%)	Normal Vitamin C (*n* = 18 100%)	Total (*n* = 39 100%)	Low Vitamin C (*n* = 21 100%)	Normal Vitamin C (*n* = 18 100%)	Total (*n* = 39 100%)	Low Vitamin C (*n* = 6 100%)	Normal Vitamin C (*n* = 8 100%)	Total (*n* = 14 100%)	Low Vitamin C (*n* = 6 100%)	Normal Vitamin C (*n* = 8 100%)	Total (*n* = 14 100%)
Microscopic AFB load												
Negative	0 (0%)	0 (0%)	0 (0.00%)	9 (42.85%)	10 (55.55%)	19 (48.72%)	0 (0.00%)	2 (25.00%)	2 (14.29%)	4 (66.67%)	6 (75.00%)	10 (71.43%)
1–9 AFB	1 (4.76%)	1 (5.55%)	2 (5.13%)	2 (9.52%)	1 (5.55%)	3 (7.69%)	0 (0.00%)	1 (12.50%)	1 (7.14%)	1 (16.67%)	0 (0.00%)	1 (7.14%)
1+	2 (9.52%)	7 (38.89%)	9 (23.08%)	9 (42.85%)	7 (38.89%)	16 (41.02%)	1 (16.67%)	2 (25.00%)	3 (21.43%)	1 (16.67%)	2 (25.00%)	3 (21.43%)
2+	9 (42.85%)	8 (44.44%)	17 (43.59%)	1 (4.76%)	0 (0.00%)	1 (2.56%)	4 (66.67%%)	3 (37.50%)	7 (50.00%)	0 (0%)	0 (0%)	0 (0%)
3+	9 (42.85%)	2 (11.11%)	11 (28.20%)	0 (0%)	0 (0%)	0 (0%)	1 (16.67%)	0 (0.00%)	1 (7.14%)	0 (0%)	0 (0%)	0 (0%)
Microscopy+												
Culture AFB load												
Negative	0 (0%)	0 (0%)	0 (0%)	6 (28.57%)	13 (72.22%)	19 (48.72%)	0 (0%)	0 (0%)	0 (0%)	3 (50.00%)	6 (75.00%)	9 (64.23%)
1+	3 (14.28%)	9 (50.00%)	12 (30.77%)	15 (71.42%)	5 (27.78%)	20 (51.28%)	2 (33.33%)	3 (37.50%)	5 (35.71%)	3 (50.00%)	2 (25.00%)	5 (35.71%)
2+	11 (52.38%)	8 (44.44%)	19 (48.72%)	0 (0%)	0 (0%)	0 (0%)	3 (50.00%)	4 (50.00%)	7 (50.00%)	0 (0%)	0 (0%)	0 (0%)
3+	7 (33.33%)	1 (5.55%)	8 (20.51%)	0 (0%)	0 (0%)	0 (0%)	1 (16.67%)	1 (12.50%)	2 (14.29%)	0 (0%)	0 (0%)	0 (0%)

**Table 6:** Evaluation of parameters according to serum vitamin C levels. ESR, Erythrocyte Sedimentation Ratio. CRP = C-reactive protein. FIB = fibrinogen. HB = hemoglobin. WBC = white blood cell. PLT = Platelet. GOT, Glutamate Oxaloacetate Transaminase. GPT = Glutamate Pyruvate Transaminase. URE = urea. CRE = creatinine.

Parameter	Normal Vitamin C	Low Vitamin C	Total
ESR difference (mm/h) *	−48.15 ± 13.94	−58.56 ± 19.07	−53.45 ± 17.40
CRP difference (mg/L) *	−21.54 ± 9.21	−29.44 ± 12.34	−25.57 ± 11.52
FIB difference (mg/dL)	−65.73 ± 49.12	−99.7 ± 48.05	−83.04 ± 51.07
HB difference (g/dL)	0.55 ± 1.16	1.21 ± 1.32	0.88 ± 1.28
WBC difference (×10^3^/mL)	−68.27 ± 1366.38	44.78 ± 1754.86	−10.68 ± 1562.24
PLT difference (×10^3^/mL)	−46,038.46 ± 112,753.0	−78,092.59 ± 119,134.5	−62,367.92 ± 116,061.9
AST difference (U/L)	−1.73 ± 21.02	3.59 ± 24.99	0.98 ± 23.06
ALT difference (U/L)	−6.19 ± 22.65	2.26 ± 21.74	−1.89 ± 22.38
Urea difference (mg/dL)	−1.62 ± 12.01	−2.93 ± 11.21	−2.28 ± 11.51

*: significant difference (*p* < 0.05) between groups of patients with normal and low vitamin C levels.

**Table 7:** Longitudinal random-effects regression of MTB load assessed by culture from baseline to 60 days.

Culture AFB Load	Coefficient	Standard Error	z	*p* > |z|	[95% Confidence Interval]	
Low vitamin C	3.83	1.81	2.12	0.034 *	0.29	7.38
Gender	0.39	2.04	0.19	0.848	−3.61	4.39
Smoking	2.88	1.92	1.50	0.134	−0.89	6.65
Underweight	0.71	1.82	0.39	0.698	−2.86	4.28
Constant	7.33	2.14	3.42	0.001 *	3.13	11.53

*: significant *p* value (<0.05).

## Data Availability

The data presented in this study are available upon request from the corresponding author. The data are not publicly available due to the patients’ personal data protection policy of the University and Hospital.
